# A Fusion-Based Machine Learning Approach for the Prediction of the Onset of Diabetes

**DOI:** 10.3390/healthcare9101393

**Published:** 2021-10-18

**Authors:** Muhammad Waqas Nadeem, Hock Guan Goh, Vasaki Ponnusamy, Ivan Andonovic, Muhammad Adnan Khan, Muzammil Hussain

**Affiliations:** 1 Faculty of Information and Communication Technology (FICT), Universiti Tunku Abdul Rahman (UTAR), Kampar 31900, Perak, Malaysia; waqasnadeem@utar.my (M.W.N.); gohhg@utar.edu.my (H.G.G.); vasaki@utar.edu.my (V.P.); 2 Department of Electronic & Electrical Engineering, University of Strathclyde, Royal College Building, 204 George St., Glasgow G1 1XW, UK; 3 Pattern Recognition and Machine Learning Lab, Department of Software, Gachon University, Seongnam 13557, Korea; 4 Department of Computer Science, School of Systems and Technology, University of Management and Technology, Lahore 54000, Pakistan; muzammil.hussain@umt.edu.pk

**Keywords:** diabetes prediction, machine learning, support vector machines, artificial neural networks, data fusion, healthcare applications, intelligent system

## Abstract

A growing portfolio of research has been reported on the use of machine learning-based architectures and models in the domain of healthcare. The development of data-driven applications and services for the diagnosis and classification of key illness conditions is challenging owing to issues of low volume, low-quality contextual data for the training, and validation of algorithms, which, in turn, compromises the accuracy of the resultant models. Here, a fusion machine learning approach is presented reporting an improvement in the accuracy of the identification of diabetes and the prediction of the onset of critical events for patients with diabetes (PwD). Globally, the cost of treating diabetes, a prevalent chronic illness condition characterized by high levels of sugar in the bloodstream over long periods, is placing severe demands on health providers and the proposed solution has the potential to support an increase in the rates of survival of PwD through informing on the optimum treatment on an individual patient basis. At the core of the proposed architecture is a fusion of machine learning classifiers (Support Vector Machine and Artificial Neural Network). Results indicate a classification accuracy of 94.67%, exceeding the performance of reported machine learning models for diabetes by ~1.8% over the best reported to date.

## 1. Introduction

Diabetes (DB) is a damaging health condition placing a significant treatment cost burden on health service providers throughout the world. Beta cells in the pancreas produce an insufficient amount of insulin with the resultant deficiency causing high levels of glucose within the blood, classified as Type-1 DB (hyper-glycemia); in Type-2, the body is unable to utilize the available insulin [1]. Moreover, DB gives rise to other clinical complications such as neurological damage, retinal degradation, and kidney and heart disease [2].

The treatment of DB is also an escalating challenge as more than 422 million adults suffered from the condition in 2014 compared to 108 million in 1980; the ratio of people-with-diabetes (PwD) referenced to the total adult population increased from 4.7% to 8.5% over the same period. Furthermore, 1.6 million diabetic patients died in 2015, and in 2012, 2.2 million further deaths were attributed to high blood glucose levels [3]. Projections indicate that DB will be the 7th major illness condition causing deaths in the global population by 2030 [4]. The timely identification and the early detection of the onset of diabetes are, therefore, of potential value in the goal of optimizing treatment pathways, providing a better quality of life for PwD, and reducing the number of deaths owing to the condition. Moreover, a significant number of PwD remain unaware of the condition until a serious complication event [4]; delays in the diagnosis of Type-2 DB during the early stages of onset increases the risk of serious complications [1,4].

A range of Machine Learning (ML) methods such as Logistic Adaptive Network-based Fuzzy Inference System (LANFIS) [5], Q-learning Fuzzy ARTMAP (FAM), Genetic Algorithm (GA) (QFAM-GA) [6], Hybrid Prediction Model (HPM) [7], Artificial Neural Network (ANN), and Bayesian Networks (BN) (ANN-BN) [8] have been used to develop algorithms for the classification of DB [9,10]. However, reported machine learning-based solutions have been limited in the accuracy of prediction, owing primarily to the lack of the required scope and volume of data for the training and testing of models. Intuitively, algorithms generated from large datasets yield more accurate performance as compared to models trained with a small number of instances of the target output. Poorly performing machine learning algorithms increase the barrier to adoption in operational settings, and thus, there is a clear need to enhance the prediction accuracy given the limited availability and accessibility of the required scope of data [11,12,13,14,15,16].

Here, core elements of a decision support system founded on the fusion of machine learning algorithms are reported for the identification of diabetes. The proposed architecture comprises multiple layers segmented as Data Sources, Data Fusion, and Fusion of Machine Learning techniques [17]. The Data Source layer receives and stores multiple streams of data from different sources such as Electronic Health Records (EHR) in a format suitable as an input to the development of the machine learning algorithms. Subsequently, the Data Fusion layer fuses data from the Data Source layer, storing the outputs on a centralized database. K fold validation is then applied for the selection of hyper-parameters, inputs to the training of the classification model. In the development reported here, Support Vector Machine (SVM) and Artificial Neural Network (ANN) machine learning classifiers have been fused using the posteriori probability method for the classification of diabetes.

A range of performance metrics, viz. Accuracy, Specificity, Sensitivity, and Precision, are used in the evaluation of the performance of the proposed approach. Diabetic datasets reported in [18,19] are used for the training and testing of the models.

The contributions of the reported research can be summarized as:

- A fusion-based machine learning architecture for the prediction of diabetes has been proposed.
- Two machine learning classifiers Support Vector Machine (SVM) and Artificial Neural Network (ANN) within the architecture have been evaluated.

The remainder of the paper is organized as follows. Section 2 summarizes related research, Section 3 describes the methodology of the proposed architecture, the theoretical and mathematical background of the selected machine learning classifiers, and the approach to their fusion are also presented in this section. The details of the evaluation of the performance of the approach are mapped and discussed in Section 4, and conclusions are drawn in Section 5.

## 2. Related Research

Artificial Neural Networks (ANN) and General Regression Neural Networks (GRNN) have been used to create algorithms for the diagnosis of diabetes [20]; the GRNN approach achieved an ~80% prediction accuracy, an improvement compared to Radial Basis Function (RBF) and Multi-Layer Perceptron (MLP) based techniques. Temurtas et al. [21] reported on a Multilayer Neural Network (MNN) implementation deploying a probabilistic Neural Network and Levenberg–Marquardt Algorithm (LMA). A two-stage approach proposed in [22] achieved a ~89.5% prediction accuracy; in Stage-I, a Principal Component Analysis (PCA) algorithm is applied to reduce the dimension of input features and in Stage-II, an Adaptive Neuro-Fuzzy Inference System generates the model. Further, an Adaptive Network-based Fuzzy System (ANFS) and Levenberg–Marquardt Algorithm (LMA)-based solution achieved an 82.3% prediction accuracy [23]. Rohollah et al. report on a Logistic Adaptive Network-based fuzzy system with 88% predictive accuracy [5] and Kemal et al. [24] developed a Least Square Support Vector Machine (LS-SVM) and Generalization Discriminant Analysis (GDA)-based cascade learning system. A k-means clustering approach reported by Bankat et al. [7] successfully eliminates incorrect samples from the dataset. Bayesian Network (BN) based diagnosis achieved a 72.3% prediction accuracy [25], whilst a three-stage diagnosis system presented by Muhammad et al. [26] uses a Genetic Algorithm (GA); several rule-based classification systems have been developed by the same research team. The rule-based system of Wiphada et al. [27] comprises two stages; in the first stage, the nodes of a neural network are pruned to determine their maximum weights; in the second stage, the data are analyzed to identify the frequency content, and then linguistic rules are created based on frequency intervals. The rule-based system has a 74% prediction accuracy. Mostafa et al. [28] present a Recursive Rule Extraction (Re-Rx) framework to generate decision rules, achieving 83.8% accuracy. In [6], a two-stage hybrid model was presented for decision rule extraction and classification. In stage-1, fuzzy logic with Q-learning is used to create decision rules and in stage-2, a Genetic Algorithm (GA) is used for the extraction of rules. Mohammad et al. [29] present a combination of Support Vectors Regression (SVR) and an ANN-based model for the detection of diabetes with 86.13% accuracy. A Gaussian Hidden Markov Model (GHMM) technique is applied in [30], achieving 85.69% accuracy; a Gaussian Hidden Markov Model (GHMM) reported in [31] achieved 85.9% accuracy; and a Deep Extreme Learning Machine (DELM) based prediction model is presented in [32] with 92.8% accuracy. A summary of the related research is presented in Table 1.

In summary, a significant body of research has been reported over the recent past detailing a range of machine learning approaches for the identification of diabetes and prediction of the onset of critical episodes in PwD. Informed by the reported advances to date, the architecture detailed here implements a fusion-based approach to improve the prediction accuracy.

## 3. Materials and Methods

### 3.1. Datasets

Two datasets are used in the training and testing of the proposed fusion-based machine learning architecture. The first dataset is derived from the publicly available National Health and Nutrition Examination Survey (NHANES) [18], consisting of 9858 records and 8 features. The second “Pima Indian diabetes ” [19] is acquired from the online repository “Kaggle”, which comprises 769 records and 8 features. Both datasets, consisting of the same features but comprising a different number of records, are detailed in Table 2. Thus, the fused dataset has 10,627 records with 8 features with an age distribution between 21–77 years. The binary response attribute takes the values ‘1’ or ‘0’, where ‘0’ means a non-diabetic patient and ‘1’ means a diabetic patient. There are 7071 (66.53%) cases in class ‘0’ and 3556 (33.46%) cases in class ‘1’.

### 3.2. System Architecture

**Figure 1:**
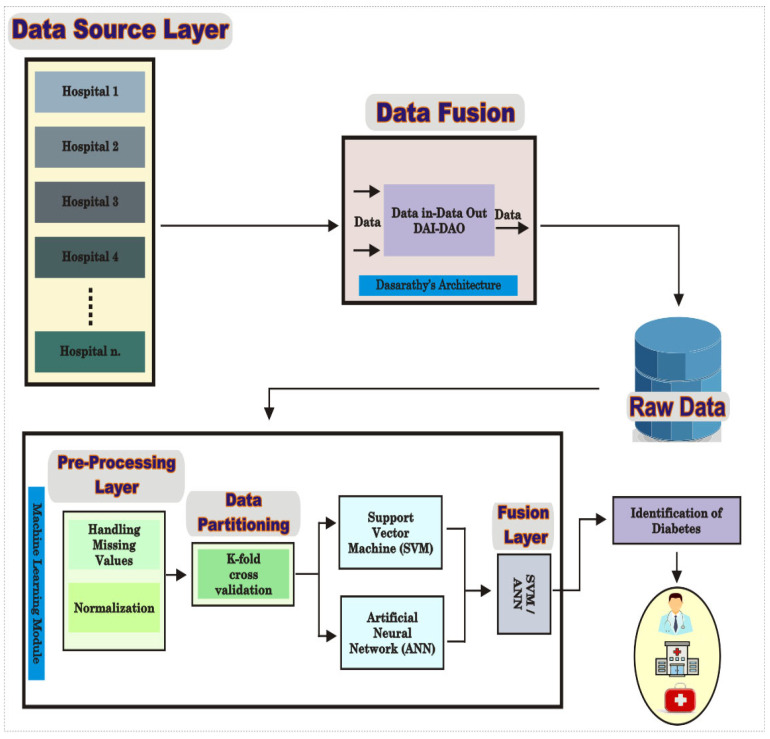
The System Architecture of the Proposed Fusion-based Prediction Approach.

The architecture consists of the following layers designated as ‘Data Source’, ‘Data Fusion’, ‘Pre-processing’, ‘Application’, and ‘Fusion’. The end-to-end process flow is described in Table 3, and the system architecture is depicted in Figure 1. The following is the methodology for the development of the algorithm.

#### 3.2.1. Data Fusion

Data Fusion is a process of association and combination of data from multiple sources [15,34], characterized by continuous refinements of its estimates, evaluation of the need for additional data, and modification of its process to achieve improved data quality. Hall et al. [35] state that the fusion of data enables the development of methods for the semi-automatic or automatic transformation of multiple sources of information from different locations and times to support effective decision-making.

Several methods such as Luo and Key [36] and Dasarathy [37] have been reported in recent years and here, the latter has been selected as it has been proven to be the most efficient in fusing data [37]. Data In-Data Out (DAI-DAO), the most elementary function in the fusion process, accepts data from the input layer and cleans the data to be more aligned to the needs of the development of machine learning algorithms.

Following the Dasarathy approach, Equation (1) represents the different data blocks $x_{1},x_{2},x_{3}\ldots \ldots \ldots x_{n}$ and the output X:

(1) $$Set\;X=\left\{x_{1},x_{2},x_{3}\ldots \ldots \ldots x_{n}\right\}$$

The degree of support, A, is indicated by the proposition of Basic Probability Assignment (BPA); the greater the BPA, the greater the degree of support for *A* (Equation (2)). The combination of different BPAs is used to reach decisions on the optimum fusion of data:

(2) $$m(X)=m_{1}⊗m_{2}⊗m_{3}\ldots \ldots ⊗m_{n}=\{\frac{1}{1-k}\sum A_{1}\cap A_{2}\cap A_{3}\ldots \ldots \cap A_{n}=X^{m_{1}\left(A_{1}\right)m_{2}\left(A_{2}\right)\ldots m_{n}\left(A_{n}\right)}\}$$

The probability of conflict—referred to as the minimum distance between data points—is captured in Equation (3), where *K* represents the probability of conflict, which is computed as:

(3) $$K=\underset{A_{1}\cap A_{2}\cap A_{3}\ldots \ldots \cap A_{n}=\emptyset }{\sum }m_{1}\left(A_{1}\right)m_{2}\left(A_{2}\right)\ldots m_{n}\left(A_{n}\right)$$

#### 3.2.2. Pre-Processing

Pre-processing is initiated by the treatment of missing values (*P*) followed by standardization (*S*). Missing or null values are imputed; otherwise, the accuracy of prediction of the machine learning classifier is compromised [32]. Here, the mean method—instead of dropping—is used to fill the missing values, formulated as in Equation (4):

(4) $$P\left(x\right)=\{\begin{array}{cc} mean\left(x\right), & if\;x=null/missing\\ x, & otherwise \end{array}$$

where x is the instances of the feature vector, which lies in n-dimensional space. The imputation of missing values by the mean method is warranted as it produces the required continuous data for the training of the algorithm without introducing outliers.

Standardization or Z-score normalization is used to rescale features, in so doing achieving a standard normal distribution with unit variance and zero mean. Standardization (*S*), formulated as in Equation (5), also reduces the skewness of the data distribution:

(5) $$S\left(x\right)=\frac{x-x^{-}}{\propto }$$

where x is the n-dimensional instances of the feature vector, $x\in R^{n}$; $x^{-}\in R^{n}$ and $\propto \in R^{n}$ are the standard deviation and mean of attributes.

#### 3.2.3. Cross-Fold Validation

The K-fold Cross-Validation (KCV) is a common approach used for model selection, error estimation of the classifiers, and splitting of data [38]. The dataset is partitioned into 5-folds; K-1 folds are used for the training and the fine-tuning of the hyper-parameters in the inner loop where the grid search algorithm was deployed [39]. In the outer loop (*k* times), the test data and optimum hyper-parameters were used for the evaluation of the model. The raw dataset contains the imbalanced ratio of negative and positive samples; a stratified KCV [40] has been used to maintain the same percentage of the samples for each class as in the original percentage. Equation (6) is used for the estimation of the final performance:

(6) $$M=\frac{1}{K}\overset{K}{\underset{n=1}{\sum }}P_{n}\pm \sqrt{\frac{\sum_{n=1}^{K}{\left(P_{n}-P^{-}\right)}^{2}}{K-1}}$$

where *M* is designated as the final performance metric for the classifier and $P_{n}\in R\;n=1,2,3,\ldots \ldots K$ is the performance metric for each fold.

#### 3.2.4. Support Vector Machines

The SVM algorithm has been used extensively for classification, regression, identification, density estimation, and time series analysis. SVM models segment the data into different groups comprising data points with similar properties. Furthermore, the fundamental principle of SVMs is to compute the optimal hyper-planes that generate the best generalization of the dataset [41,42,43]. For example, in a linear SVM model, the inputs are separated from the non-linear mapping into a high-dimensional space. The linear model constructs a new space, which represents a nonlinear decision limit between the original and the new space.

The SVM model predicts the classes for a new sample. Given a training dataset $S=\left\{\left(x_{1},\;y_{1}\right),\ldots ,\left(x_{n},\;y_{n}\right)\right\},\;x_{i}\in R_{n}$ and *y* {+1,−1} where *x_i_* represents the transferred input vector and *y_i_* the target value, the SVM becomes a binary classifier in which the class labels feature only two values +1 or −1. SVM draws an optimal hyper-plane H that separates the data into different classes and the hyper-plane H from the inputs. The objective function has convexity, a significant advantage as the solution of a quadratic programming problem and the training of SVMs are equivalent, yielding a unique solution. In contrast, the Artificial Neural Network (ANN) method requires nonlinear optimization, which may result in the algorithm being held hostage to local minimums.

The precision of the SVM algorithm is greater than other reported forecasting methods. The SVM minimizes the structural risk, while other machine learning methods focus on empirical risk minimization. In other words, the SVM method focuses on minimizing the upper limit of the generalization error to reduce the training error. SVMs process a large volume of data efficiently without overfitting. The SVM method also emphasizes the establishment of optimal hyperplanes for the separation of data. The training points ($x_{i}\to y_{i}$) that are closet to the optimal hyperplanes are referred to as support vectors and also develop the limit of the decision planes. In general, in cases when the data are not separated linearly, the SVM method uses non-linear machines to trace the optimal hyperplanes that reduce the error rate in the training set of the data [44]. The core of the SVM method theory for the solving of binary classification is described in [42,43].

Consider a set of training points, $D={\left\{x_{i},y_{i}\right\}}_{i=1}^{N}$, where the input vectors are $x_{i}=(x^{\left(1\right)},\ldots \ldots .x^{\left(n\right)})\;Ɛ\;R^{n}$ and output vectors $y_{i}\;Ɛ\;\left\{0,1\right\}$, and where *n* represents the amount of training data. Then, the optimal hyperplane used to separate the classes of data points and these optimal hyperplanes are identified by solving the following optimization problem:

(7) $$\underset{w,b}{Min}\left(\frac{1}{2}w^{t}w\right)$$

$$Subject:y_{i}(w^{t}\Phi (x_{i})+b\geq 1),\;i=1,2,3,\ldots \ldots .,n$$

where w is the Wright vector and b represents a bias variable. The non-linear function $\Phi \left(.\right):R^{n}\to R^{nk}$ maps the given inputs into a high dimensional space.

However, numerous classification problems are linearly non-separable; thus, ${£}_{i}$ denotes a gap variable used for misclassification. Hence, the optimization problem with the gap variable is written as:

(8) $$\underset{w,b,£}{Min}\left(\frac{1}{2}w^{t}w+C\overset{n}{\underset{i=1}{\sum }}{£}_{i}\right)$$

$$Subject:\{\begin{array}{ll} y_{i}\left((w^{t}\Phi (x_{i}\right)+b))+ & {£}_{i}\geq 1,\;i=1,2,3,\ldots \ldots .,n\\ & {£}_{i}\geq 0,\;i=1,2,3,\ldots \ldots .,n \end{array}$$

where *C* is used as a penalty variable for the error.

The Lagrangian construction function is used to solve the primary problem, and linear equality bound constraints are used to convert the primal into a quadratic optimization problem:

$${\mathrm{Max}}_{a}\left(\overset{N}{\underset{i=0}{\sum }}a_{i}-\frac{1}{2}\overset{n}{\underset{i=0}{\sum }}\overset{n}{\underset{j=0}{\sum }}a_{i}a_{j}Q_{\mathrm{ij}}\right)$$

$$\mathrm{Subject}:\{\begin{array}{l} 0\leq a_{i}<C,\;i=1,2,3,\ldots \ldots .,n\\ \sum_{i=0}^{N}a_{i}y_{i}=0 \end{array}$$

where $a_{i}$ is known as Lagrange multiplier $Q_{ij}=y_{i}y_{j}\;\Phi {\left(x_{i}\right)}^{t}\Phi \left(x_{j}\right)$.

The kernel function not only replaces the internal product but also satisfies the Mercer condition $K_{\left(x_{i},x_{j}\right)}=\Phi {\left(x_{i}\right)}^{t}\Phi \left(x_{j}\right),$ used for the representation of proximity or similarity between data points. Finally, the non-linear decision function is used in the primal space for the linearly non-separable case:

$$y\left(x\right)=\mathrm{sgn}\left(\sum_{i=0}^{N}a_{i}y_{i}K\left(x_{i,\;}x_{j}\right)+b\right)$$

The kernel function maps input data into a large dimensional space, where hyper-planes separate the data, rendering the data linearly separable. Different kernel functions are potential candidates for use by the SVM method:

(i) Linear Kernel: $K\left(x_{i},x_{j}\right)=x_{i}^{T}x_{j}$
(ii) Radical Kernel:$\;K\left(x_{i},x_{j}\right)=exp(-\gamma |\left|x_{i}-x_{j}\right|{|}^{2})$
(iii) Polynomial Kernel: $K\left(x_{i},x_{j}\right)={\left(yx_{i}^{T}x_{j}+r\right)}^{d}$
(iv) Sigmoid Kernel: $K\left(x_{i},x_{j}\right)=\tanh\left(\gamma x_{i}^{T}x_{j}+r\right),$ where r,d∈N and $\gamma \in R^{+}$ all are constants.

The kernel functions play an important role when the complex decision limits are defined between different classes. The selection of the decision limits is critical and challenging; hence, the selection of potential mappings is the first task for a given classification problem. The optimal selection of the potential mapping minimizes generalization errors.

In the reported research, the Radial Basis Function (RBF) kernel is selected most often for the creation of a high dimensional space for the non-linear mapping of samples. Furthermore, the RBF kernel treats non-linear problems more easily as compared to the Linear kernel. The Sigmoid kernel is not valid for some parameters.

The second challenge is the selection of hyperparameters that impact the complexity of the model. The Polynomial kernel has more hyperparameters as compared to the RBF kernel, but the latter is less computationally intensive during the Polynomial kernel, requiring more computational time at the training phase.

#### 3.2.5. Artificial Neural Networks

Artificial Neural Networks (ANNs) are inspired by the structure and functional aspects of the human biological neural system. The ANN method originates from the field of computer science, but the applications of ANNs are now widely used within a growing number of research disciplines [45]; the combination of large amounts of unstructured data (‘big data’) coupled to the versatility of the ANN architecture have been harnessed to obtain ground-breaking results in numerous application domains including natural language processing, speech recognition, and detection of autism genes. ANNs comprises many groups of interconnected artificial neurons executing computations through a connectionist approach.

Typical ANN architectures are composed of three types of nodes, viz. input, hidden, and output. The former contains the explanatory parameters and the level of attributes varies from model to model. The dependent variables are contained by the output nodes and the number of output nodes depends on choice probabilities. Nodes are connected through links and the signals propagate in a forward direction. Different numerical weights are computed from the data assigned to each link. At each node, the input value of the previous node is multiplied by the weight and summed. An activation function is used to propagate the signal into the next layer; activation functions ‘SoftMax’, ‘tan-sigmoid’, and ‘purlin’ have been used commonly in ANNs architectures. The sigmoid activation function is used here. Weigh initialization, feedforward, backpropagation for error, updating weights, and bias are integral to the ANNs.

The algebraic formulation of ANNs is:

(9) $$f_{j}=b_{1}+\overset{n}{\underset{i=1}{\sum }}\left(w_{ij}{*r}_{i}\right)$$

where the $w_{ij}$ represents the weight of neurons, $r_{i}$ represents the inputs, and b is the bias. Further, the ‘sigmoid’ activation function is written as:

(10) $${ȹ}_{k}=\frac{1}{1+e^{-f_{j}}}\;\mathrm{where}\;k=1,2,3\ldots r$$

Equation (10) is used to compute the error in back-propagation:

$$E=\frac{1}{2}\underset{k}{\sum }{\left(\tau_{k}-{ȹ}_{k}\right)}^{2}$$

where the $\tau_{k}$ denotes the desired output and ${ȹ}_{k}$ represents the calculated output. Thus, the rate of change in weights is calculated as:

$$∆w\;\propto -\frac{\partial E}{\partial w}$$

$$∆\upsilon_{j,k}=-\epsilon \frac{\partial E}{\partial \nu_{j,k}}$$

Equation (11) describes the updating of weights and biases between the hidden and output layers. By using the chain rule:

$$∆\upsilon_{j,k}=-\epsilon \;\frac{\partial E}{\partial {ȹ}_{k}}\times \frac{\partial {ȹ}_{k}}{\partial \psi_{k}}\times \frac{\partial \psi_{k}}{\partial \nu_{j,k}}$$

$$∆\upsilon_{j,k}=\epsilon \left(\tau_{k}-{ȹ}_{k}\right)\times {ȹ}_{k}\left(1-{ȹ}_{k}\right)\times \left({ȹ}_{j}\right)$$

$$∆\upsilon_{j,k}=\epsilon \xi_{k}{ȹ}_{j}$$

$$\xi_{k}=\left(\tau_{k}-{ȹ}_{k}\right)\times {ȹ}_{k}\left(1-{ȹ}_{k}\right)$$

$$∆w_{i,j}\propto -\left[\underset{k}{\sum }\frac{\partial E}{\partial {ȹ}_{k}}\times \frac{\partial {ȹ}_{k}}{\partial \psi_{k}}\times \frac{\partial \psi_{k}}{\partial {ȹ}_{j}}\right]\times \frac{\partial {ȹ}_{j}}{\partial \psi_{j}}\times \frac{\partial \psi_{j}}{\partial w_{i,j}}$$

$$∆w_{i,j}=-\epsilon \;\left[\underset{k}{\sum }\frac{\partial E}{\partial {ȹ}_{k}}\times \frac{\partial {ȹ}_{k}}{\partial \psi_{k}}\times \frac{\partial \psi_{k}}{\partial {ȹ}_{j}}\right]\times \frac{\partial {ȹ}_{j}}{\partial \psi_{j}}\times \frac{\partial \psi_{j}}{\partial w_{i,j}}$$

$$∆w_{i,j}=\epsilon \;\left[\underset{k}{\sum }\left(\tau_{k}-{ȹ}_{k}\right)\times {ȹ}_{k}\left(1-{ȹ}_{k}\right)\times \left(\nu_{j,k}\right)\right]\times {ȹ}_{k}\left(1-{ȹ}_{k}\right)\times r_{i}$$

$$∆w_{i,j}=\epsilon \;\left[\underset{k}{\sum }\left(\tau_{k}-{ȹ}_{k}\right)\times {ȹ}_{k}\left(1-{ȹ}_{k}\right)\times \left(\nu_{j,k}\right)\right]\times {ȹ}_{j}\left(1-{ȹ}_{j}\right)\times r_{i}$$

$$∆w_{i,j}=\epsilon \;\left[\underset{k}{\sum }\xi_{k}\left(\nu_{j,k}\right)\right]\times {ȹ}_{j}\left(1-{ȹ}_{j}\right)\times r$$

$$∆w_{i,j}=\epsilon \xi_{j}{\;r}_{i}$$

where

(11) $$\xi_{j}=\left[\underset{k}{\sum }\xi_{k}\left(\nu_{j,k}\right)\right]\times {ȹ}_{j}\left(1-{ȹ}_{j}\right)$$

Similarly, Equation (12) describes the updating of weight and bias between hidden and input layers:

$$\nu_{j,k}^{+}=\nu_{j,k}+\lambda_{F}∆\upsilon_{j,k}$$

(12) $$w_{i,j}^{+}=w_{i,j}+\lambda_{F}∆w_{i,j}$$

where $\lambda_{F}$ represents the learning rate.

#### 3.2.6. Fusion of SVM-ANN

Traditional machine learning classifiers can be fused by different methods and rules [14]; the most commonly used fusion rules are ‘min’, ‘mean’, ‘max’, and ‘product’ [13]. $\;P_{i}(\varphi_{j}|x)$ represents the posteriori probability, most often applied to view the output of the classifiers, and it can also be used for the implementation of fusion rules. $P_{i}$ represents the output of the *i*^th^-classifier, $\varphi_{i}$ represent the *i*^th^-class of objects, and $P_{i}(x|\varphi_{j})$ represents the probability of *x* in the *j*^th^-classifier given that the *j*^th^-class of objects occured. As the proposed objective of the architecture is a two-class output, the posteriori probability can be written as:

$$P_{i}(\varphi_{j}\left|x\right)=\frac{P_{i}(x|\varphi_{j})P(\varphi_{j})}{P_{i}\left(x\right)}$$

$$P_{i}(\varphi_{j}\left|x\right)=\frac{P_{i}(x|\varphi_{j})P(\varphi_{j})}{P_{i}(x|\varphi_{1})P(\varphi_{1})+P_{i}(x|\varphi_{2})P(\varphi_{2})}$$

j=1,2 and i=1,2,3……,L

where *L* represents the number of classifiers; here, 2 classifers are selected, SVM & ANN. Thus, the posteriori probability for the target class can be written as:

(13) $$P_{i}(\varphi_{t}\left|x\right)=\frac{P_{i}(x|\varphi_{t})P(\varphi_{t})}{P_{i}(x|\varphi_{t})P(\varphi_{t})+\theta_{i}\cdot P(\varphi_{o})}$$

where $\varphi_{t}$ represents the target class, $\varphi_{o}$ is the outlier class, and $\theta_{i}$ is the uniform distribution of density for the feature set, and where $P(\varphi_{t})$, $P(\varphi_{o}),$ and $P_{i}(x|\varphi_{t})$ represent the probability of the target class, probability of the outlier class/miss predicted class, and probability of event *x* in the *i*^th^-classifier given that the target class of object has occurred, respectively.

In the architecture, the mean fusion rule is applied, formulated as:

$$\mu (\varphi_{t}\left|x\right)=\frac{1}{L}\overset{L}{\underset{i=0}{\sum }}P_{i}(\varphi_{t}\left|x\right)$$

$$\mu (\varphi_{o}\left|x\right)=\frac{1}{L}\overset{L}{\underset{i=0}{\sum }}P_{i}(\varphi_{o}\left|x\right)$$

where $P_{i}(\varphi_{t}|x)$ and $P_{i}(\varphi_{o}|x)$ represent the probabilty of the target class given that *x* event has occurred in the *i*^th^-classifier and the probabilty of the outlier class given that *x* event has occurred in the *i*^th^-classifier. Then, the decision criteria are computed as:

$$\mu (\varphi_{t}\left|x\right)<\mu (\varphi_{o}|x)\;\mathrm{where}\;x\;\mathrm{is}\;\mathrm{an}\;\mathrm{outlier}$$

For *j*^th^-class, the fusion rule can be written as:

$$\mu (\varphi_{t}\left|x\right)=\frac{1}{L}\overset{L}{\underset{i=0}{\sum }}P_{i}(\varphi_{t}\left|x\right)$$

After substitues, the values of $P_{i}(\varphi_{t}|x)$ are

$$\mu (\varphi_{t}\left|x\right)=\frac{1}{L}\overset{L}{\underset{i=0}{\sum }}\frac{P_{i}(x|\varphi_{t})P(\varphi_{t})}{P_{i}\left(x\right)}$$

If $P_{i}\left(x\right)≅P\left(x\right)\forall_{i}$ then the above can be written as:

$$\mu (\varphi_{t}\left|x\right)=\frac{1}{L}\overset{L}{\underset{i=0}{\sum }}\frac{P_{i}(x|\varphi_{t})P(\varphi_{t})}{P\left(x\right)}$$

$$\mu (\varphi_{t}\left|x\right)=\frac{1}{L}\frac{P(\varphi_{t})}{P\left(x\right)}\overset{L}{\underset{i=0}{\sum }}P_{i}(x|\varphi_{t})$$

where,

$$Y_{\mathrm{avg}}\left(x\right)=\frac{1}{L}\overset{L}{\underset{i=0}{\sum }}P_{i}(x|\varphi_{t})$$

The target output is then computed as follows:

(14) $${\;\theta }^{-}=\frac{P\left(\varphi_{o}\right)}{P\left(\varphi_{t}\right)}\cdot \frac{1}{L}\overset{L}{\underset{i=0}{\sum }}\theta_{i}$$

The decision criteria, therefore, simplify as $Y_{avg}\left(x\right)<\theta^{-}$, where *x* is an outlier. Equation (14) represents the density function used to combine the class-conditional probability instead of posterior probabilities, which are estimated by each classifier. $Y_{avg}\left(x\right)$ is the final output of the architecture, and $\theta^{-}$ is used as a threshold that can be independently tuned to attain the desired trade-off between the false-negative rate and the false-positive rate.

## 4. Performance Evaluation

### 4.1. Performance Evaluation Matrix

A matrix comprising the accuracy, specificity, sensitivity, miss rate, precision, false-positive ratio, and the false-negative ratio is used to evaluate the performance of the algorithm [46]. A binary confusion matrix is used to compute the matrix. The development and evaluation of the solution has been performed in the Python 3.7 environment using a range of machine learning libraries on Intel^®^ Core™ i3-3217U CPU @ 1.80 GHz PC.

The definition of the performance parameters within the matrix are as follows:

$$\mathrm{Accuracy}\;=\frac{\mathrm{TP}+\mathrm{TN}}{\mathrm{TP}+\mathrm{TN}+\mathrm{FP}+\mathrm{FN}}\times 100\%$$

$$\mathrm{Miss}\;\mathrm{rate}\;=\frac{\mathrm{FP}+\mathrm{FN}}{\mathrm{TP}+\mathrm{TN}+\mathrm{FP}+\mathrm{FN}}\times 100\%$$

$$\mathrm{Sensitivity}=\mathrm{recall}=\frac{\mathrm{TP}}{\mathrm{TP}+\mathrm{FN}}\times 100\%$$

$$\mathrm{Specificity}\;=\frac{\mathrm{TN}}{\mathrm{TN}+\mathrm{FP}}\times 100\%$$

$$\mathrm{Precision}=\frac{\mathrm{TP}}{\mathrm{TP}+\mathrm{FP}}\times 100\%$$

$$\mathrm{False}\;\mathrm{positive}\;\mathrm{ratio}\;=1-\left(\frac{\mathrm{specificity}}{100}\right)$$

$$\mathrm{False}\;\mathrm{negative}\;\mathrm{ratio}\;=1-\left(\frac{\mathrm{sensitivity}}{100}\right)$$

### 4.2. Performance Results and Discussion

The performance of both classifiers has been evaluated as standalone and after fusion. A comparative analysis shows that the fusion of SVM and ANN provides an enhancement of the accuracy of prediction compared to the performance of SVM and ANN standalone algorithms. Results indicate a classification accuracy of 94.6%, exceeding the performance of machine learning models reported to date such as Random Forest (RF) [7] and Naïve Bayes (NB).

**Figure 2:**
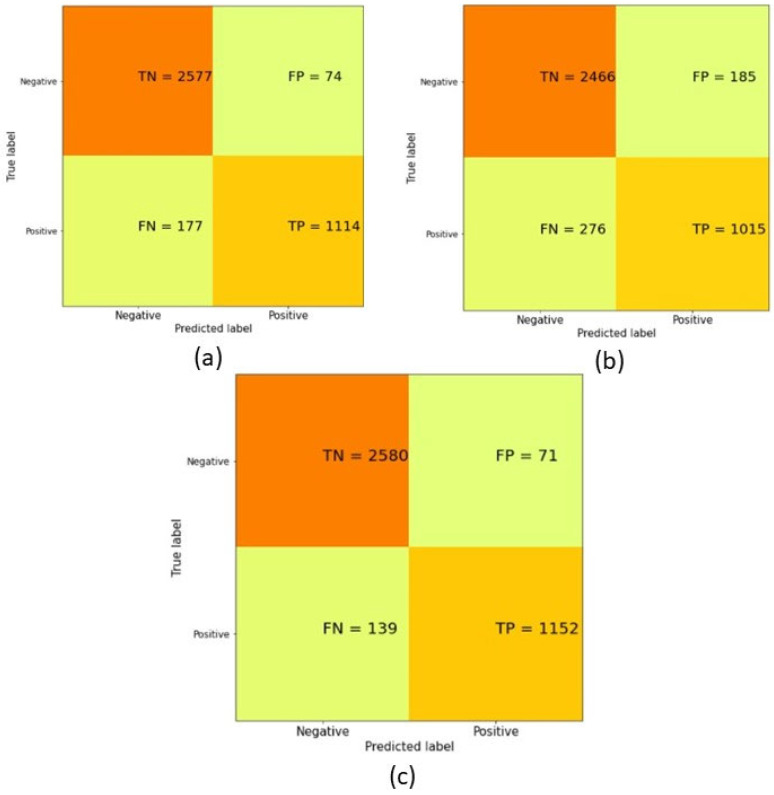
(**a**) Confusion matrix of ANN; (**b**) Confusion matrix of SVM; (**c**) Confusion matrix of Fusion (SVM-ANN).

**Figure 3:**
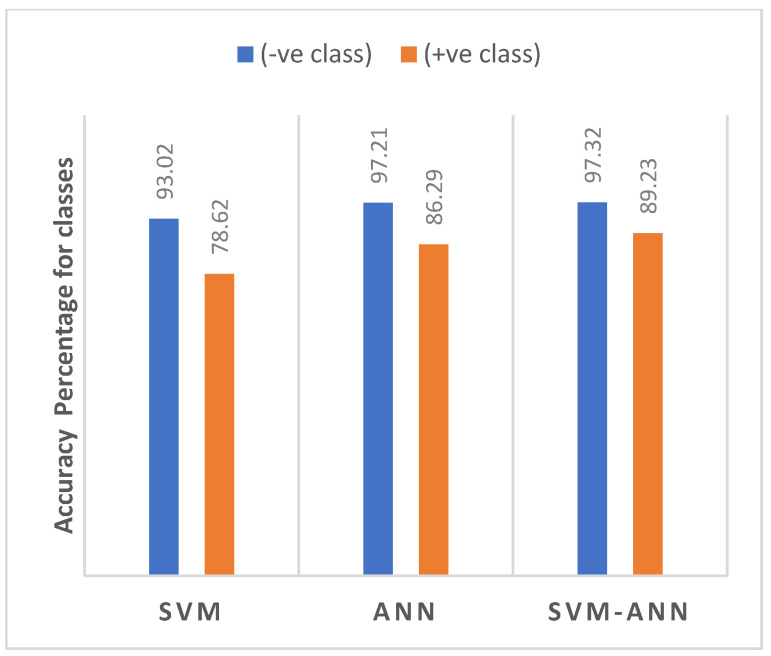
Class level Comparison of Accuracy.

Figure 2a–c show the confusion matrix of ANN, SVM, and SVM-ANN, respectively. Figure 3 shows a class level comparison of the different machine learning methods, viz. SVM, ANN, and Fusion of SVM and ANN (SVM-ANN), which are used within the architecture. Results indicate that the SVM method yields a 93.02% accuracy for the negative class (healthy) and 78.62% accuracy for the positive class (diabetic); the ANN method: 97.21% for the negative and 86.29% for the positive class; and SVM-ANN: 97.32% for the negative and 89.23% for the positive class.

The results of the comparative analysis of the performance of the architecture is also presented in Table 4. The SVM, ANN, and SVM-ANN approaches achieve 88.30%, 93.63%, and 94.67% accuracy, respectively, indicating an improvement in performance owing to the fusion of both.

**Figure 4:**
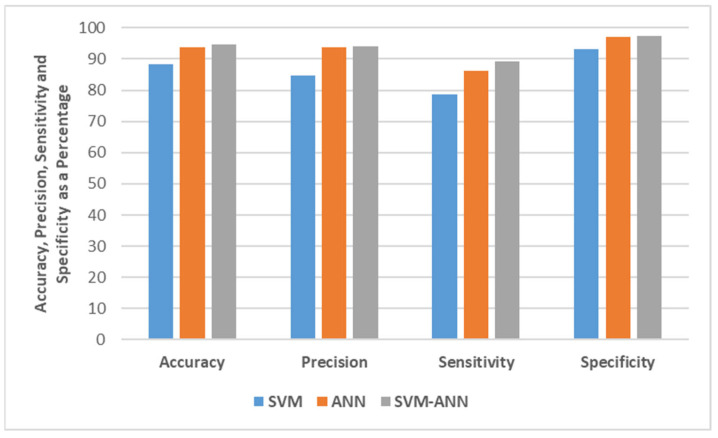
Comparison in terms of Accuracy, Precision, Sensitivity, and Specificity.

Figure 4 shows the comparison in terms of accuracy, precision, sensitivity, and specificity. On inspection, the SVM-ANN produces the best prediction in terms of accuracy in comparison with standalone SVM and ANN, improving the accuracy by a margin of 6.37% and 1.04%, respectively. The SVM-ANN model also improves the true-positive rate and also evident is that the degree of miss-classification is decreased by the SVM-ANN model. Furthermore, the fused architecture improves the system performance in terms of balanced accuracy, precision, sensitivity, and specificity (average of accuracy, precision, sensitivity, and specificity) by 3.71%, 4.98%, 6.78%, and 2.21%, respectively.

**Figure 5:**
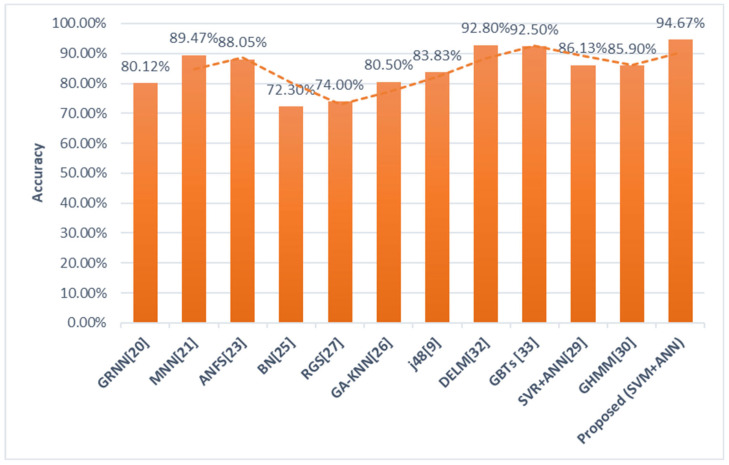
Performance Comparison of the Proposed Approach with existing Models in terms of Accuracy.

A comparison of the performance of the fusion-based system with models reported in the literature in terms of accuracy is shown in Figure 5. Results demonstrate that the architecture reported here yields an increase in the accuracy of the classification of diabetes with ~1.8% improvement compared to the best performing algorithm.

## 5. Conclusions

Machine learning methods and techniques are beginning to play an ever-increasing role in the domain of healthcare for the analysis of medical data to support the diagnosis and inform on the optimum treatment of critical health conditions. However, the existence and availability of sufficiently large datasets for the training and testing of machine learning models remain a barrier to achieving better-performing algorithms. Thus, given the prevailing restrictions in the scope and quality of available data, the paper reports on the development and evaluation of the performance of an approach based on the fusion of two machine learning methods for the prediction of diabetes.

The architecture performs data fusion to prepare a coherent dataset derived from multiple streams (locations) in order to be better aligned to the needs of the development of machine learning algorithms. The algorithms are then created through the fusion of two well-known machine learning classifiers: SVM and ANN, for the identification and prediction of the onset of critical events for PwD.

A comparison of performance with the published literature shows that the proposed architecture accuracy of 94.67% is an improvement of ~1.8% when compared to the best-performing model reported to date. In the future, other machine learning classifiers such as Random Forest, Decision Tree, and Naïve Base can also be consideredat the machine learning fusion layer.

## Figures and Tables

**Table 1:** Summary of the recent development of Machine Learning for Diabetic Prediction.

Studies	Proposed Methods	Dataset	Findings
[5]	Logistic Adaptive Network Fuzzy Inference System (LANFIS)	Pima Indians diabetes	Prediction accuracy = 88.05% Sensitivity = 92.15% Specificity = 81.63%
[7]	Hybrid Prediction Model (HPM)+ C 4.5	Pima Indian diabetes	Prediction accuracy = 92.38%
[20]	Artificial Neural Networks (ANN) + General Regression Neural Networks (GRNN)	Pima Indian diabetes	Prediction accuracy = 80%
[22]	Principal Component Analysis (PCA) + Adaptive Neuro-Fuzzy Inference System (ANFIS)	Pima Indian diabetes	Prediction accuracy = 89.47%
[23]	Adaptive Network-based Fuzzy System (ANFS) + Levenberg–Marquardt Algorithm	Pima Indian diabetes	Prediction accuracy = 82.30% Sensitivity = 66.23% Specificity = 89.78%
[24]	Least Square Support Vector Machine (LS-SVM) and Generalization Discriminant Analysis (GDA)	Pima Indian diabetes	Classification accuracy = 82.05% Sensitivity = 83.33% Specificity = 82.05%
[25]	Bayesian Network (BN)	Pima Indian diabetes	Prediction accuracy = 72.3%
[26]	(1) Genetic Algorithm (GA) + K-Nearest Neighbors (GA-KNN), (2) Genetic Algorithm (GA) + Support Vector Machine (GA-SVM)	Pima Indian diabetes	Prediction accuracy = 80.5%, Prediction accuracy = 87.0%,
[31]	Gaussian Hidden Markov Model (GHMM)	CPCSSN clinical dataset	Prediction accuracy = 85.9%
[32]	Deep Extreme Learning Machine (DELM)	Pima Indian diabetes	Prediction accuracy = 92.8%
[33]	Gradient Boosted Trees (GBTs)	Canadian AppleTree and the Israeli Maccabi Health Services (MHS)	Prediction accuracy = 92.5%
	Proposed SVM-ANN		Prediction accuracy = 94.67% Sensitivity = 89.23% Specificity = 97.32%

**Table 2:** Diabetes Datasets—Features Description.

S#	Feature Name	Description	Variable Type
1	Glucose (F1)	Plasma glucose concentration at 2 h in an oral glucose tolerance test	Real
2	Pregnancies (F2)	Number of times pregnant	Integer
3	Blood Pressure (F3)	Diastolic blood pressure (mm HG)	Real
4	Skin Thickness (F4)	Triceps skinfold thickness (mm)	Real
5	Insulin (F5)	2-h serum insulin (mu U/mL)	Real
6	BMI (F6)	Body mass index (weight in kg/(height in)^2^	Real
7	Diabetes Pedigree Function (F7)	Diabetes Pedigree Function	Real
8	Age (F8)	Age (years)	Integer

**Table 3:** Steps for the Implementation of the Proposed Architecture.

1 Begin 2 Input Data 3 Apply Data fusion technique 4 Preprocess the data by different techniques 5 Data partitioning using the K-fold cross-validation method 6 Classification of diabetes and healthy peoples using SVM and ANN 7 Fusion of SVM and ANN 8 Computes performance of the architecture using a different evaluation matrix 9 Finish

**Table 4:** Overview of Simulation Results.

Evaluation Matrix	SVM	ANN	Fusion of SVM-ANN
Accuracy	88.30%	93.63%	94.67%
Specificity	93.02%	97.20%	97.32%
Sensitivity	78.62%	86.28%	89.23%
Precision	84.58%	93.77%	94.19%
Miss rate	11.70%	6.37%	5.33%
False Positive Ratio (FPR)	0.06	0.02	0.02
False Negative Ratio (FNR)	0.21	0.13	0.10

## Data Availability

Not applicable.
